# Following de novo triglyceride dynamics in ovaries of *Aedes aegypti* during the previtellogenic stage

**DOI:** 10.1038/s41598-021-89025-6

**Published:** 2021-05-05

**Authors:** Lilian Valadares Tose, Chad R. Weisbrod, Veronika Michalkova, Marcela Nouzova, Fernando G. Noriega, Francisco Fernandez-Lima

**Affiliations:** 1 Department of Chemistry and Biochemistry, Florida International University, 11200 SW 8th St AHC4-233, Miami, FL 33199 USA; 2 National High Magnetic Field Laboratory, Florida State University, Tallahassee, FL USA; 3 Department of Biology, Florida International University, Miami, FL USA; 4 Biomolecular Science Institute, Florida International University, Miami, FL USA; 5 Institute of Parasitology, Biology Centre CAS, Ceske Budejovice, Czech Republic; 6 Institute of Zoology, Slovak Academy of Sciences, Dubravska cesta 9, 84506, Bratislava, Slovakia

**Keywords:** Biological techniques, Biophysics, Chemical biology, Developmental biology, Molecular biology, Chemistry, Analytical chemistry, Biochemistry, Chemical biology

## Abstract

Understanding the molecular and biochemical basis of egg development is a central topic in mosquito reproductive biology. Lipids are a major source of energy and building blocks for the developing ovarian follicles. Ultra-High Resolution Mass Spectrometry (UHRMS) combined with in vivo metabolic labeling of follicle lipids with deuterated water (^2^H_2_O) can provide unequivocal identification of de novo lipid species during ovarian development. In the present study, we followed de novo triglyceride (TG) dynamics during the ovarian previtellogenic (PVG) stage (2–7 days post-eclosion) of female adult *Aedes aegypti*. The incorporation of stable isotopes from the diet was evaluated using liquid chromatography (LC) in tandem with the high accuracy (< 0.3 ppm) and high mass resolution (over 1 M) of a 14.5 T Fourier Transform Ion Cyclotron Resonance Mass Spectrometer (14.5 T FT-ICR MS) equipped with hexapolar detection. LC-UHRMS provides effective lipid class separation and chemical formula identification based on the isotopic fine structure. The monitoring of stable isotope incorporation into de novo incorporated TGs suggests that ovarian lipids are consumed or recycled during the PVG stage, with variable time dynamics. These results provide further evidence of the complexity of the molecular mechanism of follicular lipid dynamics during oogenesis in mosquitoes.

## Introduction

Nutrient availability and allocation towards life processes must be in balance during the insect life cycle^1–3^. For example*,* lipid allocation involves trade-off decisions when female mosquitoes mobilize lipids towards the ovaries as a source of oocyte maturation, but they also require lipids for energy homeostasis^4–6^. There are three major periods in the ovary development during a gonotrophic cycle in *Aedes aegypti* mosquitoes: previtellogenesis (PVG), ovarian resting stage (ORS) and vitellogenesis (VG)^7,8^*.* Females emerge with 40 μm immature primary follicles that grow into 100 μm mature PVG follicles in the next 48–72 h. Oocytes remain in a dynamic “state of arrest” during the ORS, and will enter VG only after a blood meal. During the VG stage yolk protein precursors are incorporated by developing oocytes, oogenesis is completed, and the eggs are laid^9^. Lipids and glycogen are the primary energy reserves for egg development during the immature stages^5,10,11^. These teneral reserves are partially consumed during the PVG period; moreover, nectar-feeding adds critical reserves during the ORS, and a blood meal triggers VG^8,11–14^. Previous studies revealed in *Aedes aegypti*, more than 80% of lipids found in eggs originate from sugars consumed before a blood meal^15,16^.

**Figure 1:**
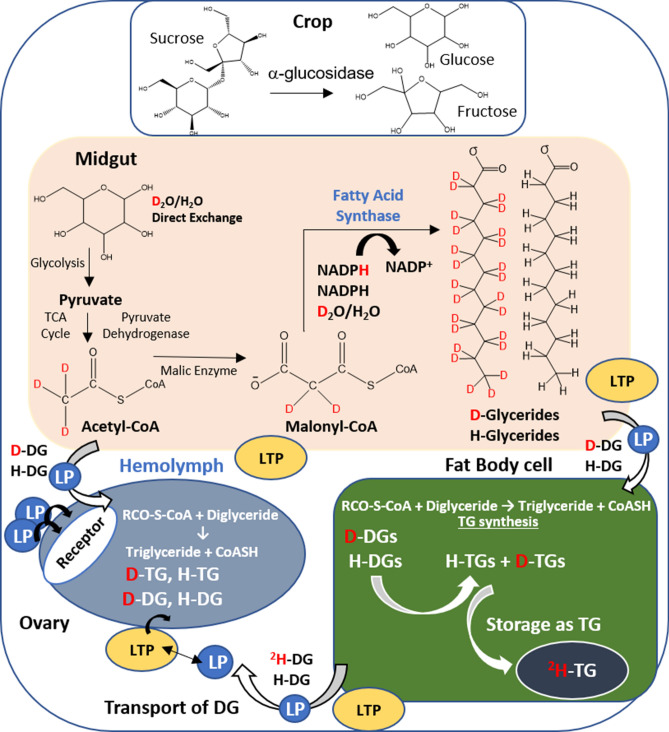
Deuterium incorporation into triglycerides occurs during de novo fatty acid synthesis. Different tissue compartments are represented (crop, midgut, hemolymph, ovary and fat body). The TG synthetic pathway utilizes substrates that have been labelled with deuterium, including acetyl CoA, NADPH and water. Hydrogen atoms (H) highlighted in red indicate the location where deuterium (D) may have replaced hydrogen in a newly synthesized molecule. *TCA cycle* tricarboxylic acid cycle, *CoASH* coenzyme A, *RCO-S-CoA* acetyl-coenzyme A, *malonyl-CoA* malonyl coenzyme A, *NADPH* nicotinamide adenine dinucleotide phosphate, *LTP* lipid transfer particle, *LP* lipophorin, *DG* diglyceride, *TG* triglyceride.

Sugar-feeding is a critical source of nutrients during the PVG stage. Sugar digestion starts in the crop, from which part of the meal is transferred periodically into the midgut (Fig. 1)^17–19^. Enzymes from the saliva ingested with the sugar meal and the midgut convert sucrose in glucose and fructose^17,20^. The biosynthesis of lipids begins in the midgut; sugars are used as precursors for fatty acid (FA) and triglycerides (TG) synthesis^21,22^. Experiments using radiolabeled sugars confirmed that they are utilized as substrate for TG synthesis^23,24^. While TG is the principal lipid produced in the fat body^25–27^, most of the lipids found in the hemolymph are in the form of diglycerides (DG)^25,26,28^. The transport of lipids among tissues is mediated by Lipophorin (Lp) and the Lipid Transfer Particle (LTP)^17,19,29,30^. Previous studies suggested that the ovaries can only synthesize small amounts of lipids^31,32^, therefore the majority of ovarian lipids must be produced elsewhere (e.g., fat body)^16,17,28,33^. While TG reserves can be carried from the larva stage, de novo TG synthesis play undoubtedly a key role in *Ae. aegypti* oogenesis^15,27,34^.

In the present work, we studied ovarian TG dynamics during the PVG stage (2–7 days post-eclosion) of sugar-fed female *Ae. aegypti*. Stable isotopes from deuterated water were incorporated into the mosquito sugar diet, and TGs were detected using liquid chromatography coupled to Ultrahigh Resolution Mass Spectrometry (LC-UHRMS)^29,35–38^. When a sugar diet with ^2^H_2_O was provided (Fig. 1), de novo synthesized TGs were labeled with ^2^H along the fatty acyl tails^39–41^. This procedure permitted the identification of de novo synthesized TGs, as well as the analysis of the dynamics of ovary TG incorporation. A 14.5 T Fourier transform ion cyclotron resonance mass spectrometer (FT-ICR MS) equipped with hexapolar detection was utilized for effective isotope separation and chemical composition assignment. TGs were classified based on the fatty acid length and number of unsaturated bonds. Their relative abundances and degree of stable isotope incorporation (e.g., deuterium) were measured as a function of the time after adult eclosion.

## Results

### De novo synthesis of ovarian lipid reserves

The use of stable isotope labelling combined with ultrahigh resolution mass spectrometry has shown significant advantages for the analysis of biological pathways^6,27,29,42,43^. Different from other labelling techniques, the incorporation of stable isotopes does not change the chemical properties of the lipid and allows the detection of the labeled molecule by their isotopic profile. This approach enables direct analysis of nutrient distribution, mobilization and metabolism^4,24,38,44–46^. While the concept is simple, for the analysis of complex biological samples it requires: (i) the use of complementary pre-separation techniques to diminish matrix effects and increase the sensitivity of the analysis^28,38,47–54^ and (ii) the use of high magnetic fields to achieve ultrahigh mass resolution with short transient duration for compatibility with LC separation (e.g., resolving power greater than m/Δm_50%_ > 1,000,000 and mass accuracy better than 1 ppm)^28,37,42–44,47–49^. More recently, in addition to the use of higher magnetic fields, alternative detection strategies (e.g., hexapolar detection, 3Ω +) has allowed better sensitivity and shorter analysis time, making more efficient the coupling of LC-FT-ICR MS^42,47,48,55^. Adult female mosquitoes were offered either 20% sucrose/water or 20% sucrose/^2^H_2_O water for a period of 2, 3, 4, 6 and 7 days after emergence. Inclusion of deuterated water (^2^H_2_O) in the mosquito diet during the PVG stage, provided unequivocal identification of de novo TG dynamics. When offered a sucrose-^2^H_2_O diet, adult female mosquitoes synthesized de novo FA using isotopically labeled substrates, such as acetyl CoA, NADPH and water, which became metabolically enriched with ^2^H (Fig. 1).

### Identification and quantification of TGs from mosquito ovaries

**Figure 2:**
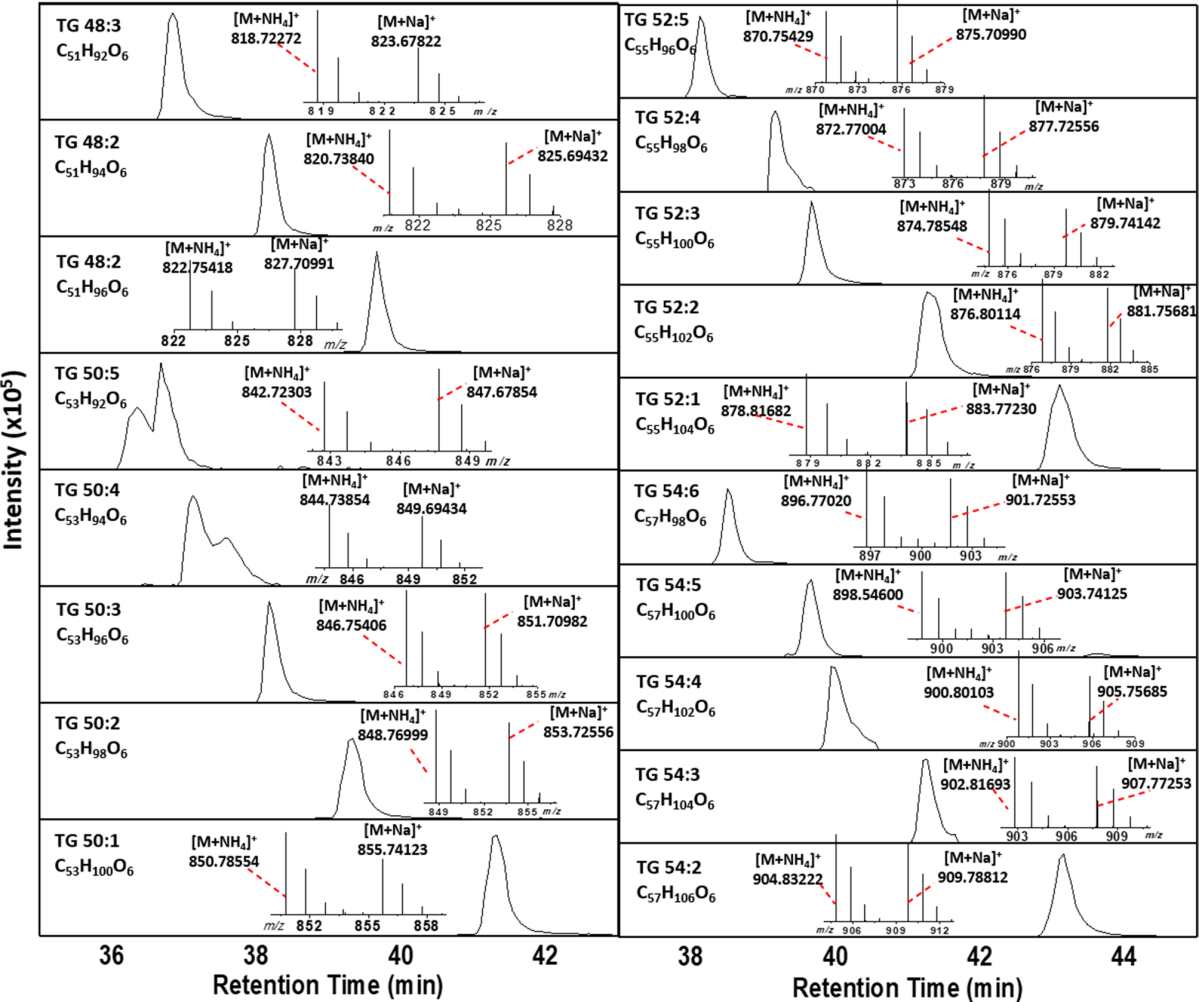
Typical extracted ion chromatograms of the [M + NH_4_]^+^ molecular ion form for all the TG species observed. In the insets, typical MS projection showing the relative abundance of the [M + NH_4_]^+^ and [M + Na]^+^ molecular ion forms.

Typical LC and MS profiles for unlabeled TGs extracted from ovaries generated using the LC-URMS workflow are shown in Fig. 2. TG signals were in the 800–900 m/z range, eluting at 35–45 min after injection of the sample. The molecular ammonium adduct ion forms ([M + NH_4_]^+^) of TG species showed higher abundance than the sodium adduct ions ([M + Na]^+^), and were used for quantitation analysis (Fig. 2). In most cases, a single LC band was observed per TG; however, in the case of TG 50:4 and TG 50:5, the double bands corresponded to the presence of positional isomers. Table 1 displays a summary of the TGs detected using LC-FT-ICR MS with a mass accuracy lower than 300 ppb. TGs extracted from ovaries of females fed ^2^H_2_O labeled sugar diets revealed similar EIC profiles; although, the addition of ^2^H creates distinct isotopic patterns (Figure S1). The use of ultrahigh resolution FT-ICR MS allowed the observance of the isotopic fine structure. This permits direct discrimination of the signals containing ^2^H from that of naturally occurring isotopes (e.g., ^13^C).

**Table 1:** LC-FT-ICR MS triglyceride assignments for ovarian TG 48, TG 50, TG 52 and TG 54.

Lipid	Neutral formula	[M + NH_4_]^+^	Theoretical mass	Experimental mass	Retention time (min)	Error (ppm)
TG 15:0/18:1(d7)/15:0	C_51_H_89_O_6_D_7_	[C_51_H_89_O_6_D_7_ + NH_4_]^+^	829.798454	829.79840	39.1–40.1	0.065
TG 48:3	C_51_H_92_O_6_	[C_51_H_92_O_6_ + NH_4_]^+^	818.723216	818.72272	37.9–38.1	0.124
TG 48:2	C_51_H_94_O_6_	[C_51_H_94_O_6_ + NH_4_]^+^	820.738866	820.73840	38.1–39.1	0.136
TG 48:1	C_51_H_96_O_6_	[C_51_H_96_O_6_ + NH_4_]^+^	822.754516	822.75418	39.6–40.3	0.109
TG 50:5	C_53_H_92_O_6_	[C_53_H_92_O_6_ + NH_4_]^+^	842.723216	842.72303	36.2–37.3	0.229
TG 50:4	C_53_H_94_O_6_	[C_53_H_94_O_6_ + NH_4_]^+^	844.738866	844.73854	37.1–38.2	0.440
TG 50:3	C_53_H_96_O_6_	[C_53_H_96_O_6_ + NH_4_]^+^	846.754516	846.75406	38.2–39.0	0.320
TG 50:2	C_53_H_98_O_6_	[C_53_H_98_O_6_ + NH_4_]^+^	848.770166	848.76999	39.7–40.5	0.250
TG 50:1	C_53_H_100_O_6_	[C_53_H_100_O_6_ + NH_4_]^+^	850.785816	850.78554	41.2–42.0	0.153
TG 52:5	C_55_H_96_O_6_	[C_55_H_96_O_6_ + NH_4_]^+^	870.754516	870.75429	38.0–38.6	0.180
TG 52:4	C_55_H_98_O_6_	[C_55_H_98_O_6_ + NH_4_]^+^	872.770166	872.77004	38.6–39.7	0.245
TG 52:3	C_55_H_100_O_6_	[C_55_H_100_O_6_ + NH_4_]^+^	874.785816	874.78548	39.7–40.4	0.105
TG 52:2	C_55_H_102_O_6_	[C_55_H_102_O_6_ + NH_4_]^+^	876.801466	876.80114	41.3–42.1	0.120
TG 52:1	C_55_H_104_O_6_	[C_55_H_104_O_6_ + NH_4_]^+^	878.817116	878.81682	43.1–43.7	0.245
TG 54:6	C_57_H_98_O_6_	[C_57_H_98_O_6_ + NH_4_]^+^	896.770166	896.77020	38.1–38.5	0.175
TG 54:5	C_57_H_100_O_6_	[C_57_H_100_O_6_ + NH_4_]^+^	898.785816	898.546	38.8–39.5	0.109
TG 54:4	C_57_H_102_O_6_	[C_57_H_102_O_6_ + NH_4_]^+^	900.801466	900.80103	40.0–40.4	0.350
TG 54:3	C_57_H_104_O_6_	[C_57_H_104_O_6_ + NH_4_]^+^	902.817116	902.81693	41.2–41.7	0.240
TG 54:2	C_57_H_106_O_6_	[C_57_H_106_O_6_ + NH_4_]^+^	904.832766	904.83222	43.0–44.2	0.170

**Figure 3:**
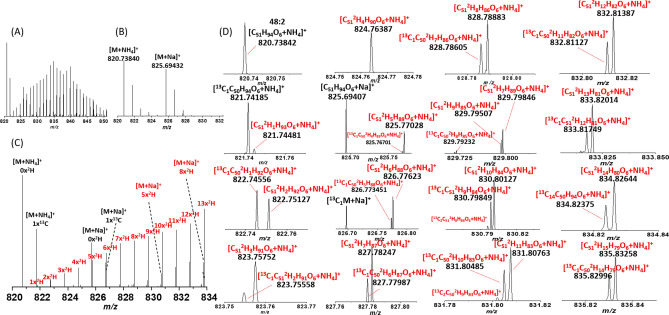
**(A,B)** Typical MS projections for the signal corresponding to 48:2 TG from ovarian samples. Deuterium labeled diet **(A)** and normal diets **(B)**. Note the increase complexity of the MS signals with increases of the number of deuteriums incorporated and ion forms. **(C,D)** Amplified MS projections denoting the sources for isobaric interferences in the 820–834 *m/z* range, and the corresponding nominal masses. The TG species containing deuterium are highlighted in red.

The de novo synthesized TGs have the characteristic presence of ^2^H in their elemental composition (Fig. 3). For example, the mass profile of de novo synthesized TG 48:2 showed the substitution of 30 hydrogens by deuterium atoms (Fig. 3A). These substitutions were absent in ovarian samples from females that received a non-labeled sucrose diet (Fig. 3B). A closer inspection to the 820–834 *m/z* range highlights the importance of ultrahigh resolution MS for this type of analysis (Fig. 3C). For example, at the nominal mass level, the 826.70 – 826.80 range contains signals from TG 48:2 [M + Na^+^] 1×^13^C isotope, TG 48:2 [M + NH_4_^+^] 5×^2^H and 1×^13^C isotopes, and TG 48:2 [M + NH_4_^+^] with 6×^2^H. These signals measured at lower resolving powers would result in convolution of the two peaks, contributing ambiguous peak assignment and spurious quantification values.

**Figure 4:**
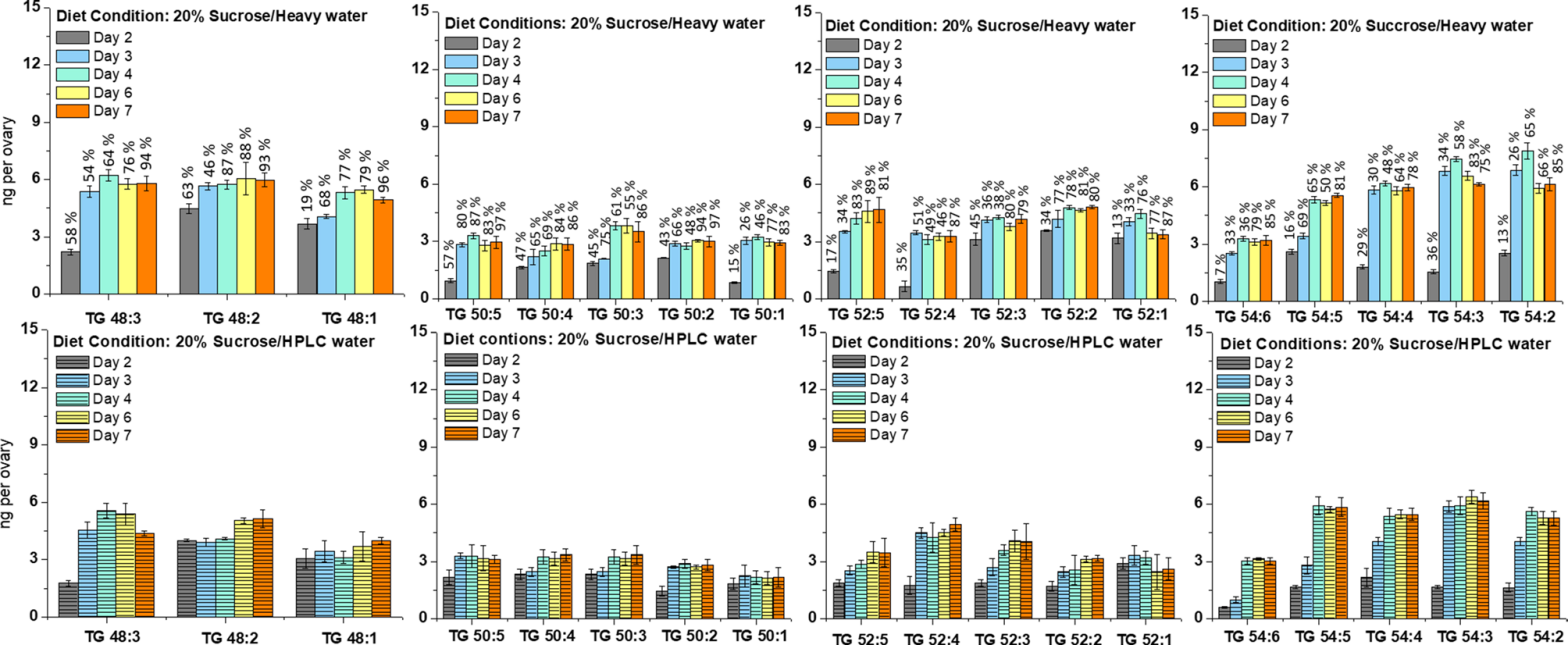
Total TG per ovary as a function of diet and time. Upper panel: 20% Sucrose/Heavy water diet. The numbers in the top are referring to the percentages of ^2^H incorporated into TGs. Lower panel: 20% sucrose/HPLC water diet. Bars are means standard error (± SE) of the analysis of triplicates samples. Each sample contained 10 ovaries.

The trends of changes and quantities of TG were similar in ovaries from females raised on non-labeled diet and labeled diet (Fig. 4). In good agreement with previous reports, TGs containing 48, 50, 52, and 54 carbons were the most abundant species^28^. As a general tendency, the amount of TGs increased with the days after eclosion, reaching a steady state towards the last days. Most significantly, deuterium incorporation was observed in all TGs species, but with unique dynamics for each of them. In terms of total lipid amounts, three-quarters of TG lipids were labeled during the six-day experiment, with the percentage of ^2^H incorporation increasing as a function of time (Figs. 4 and S2).

### Dynamics of de novo TG incorporation into the ovary

**Figure 5:**
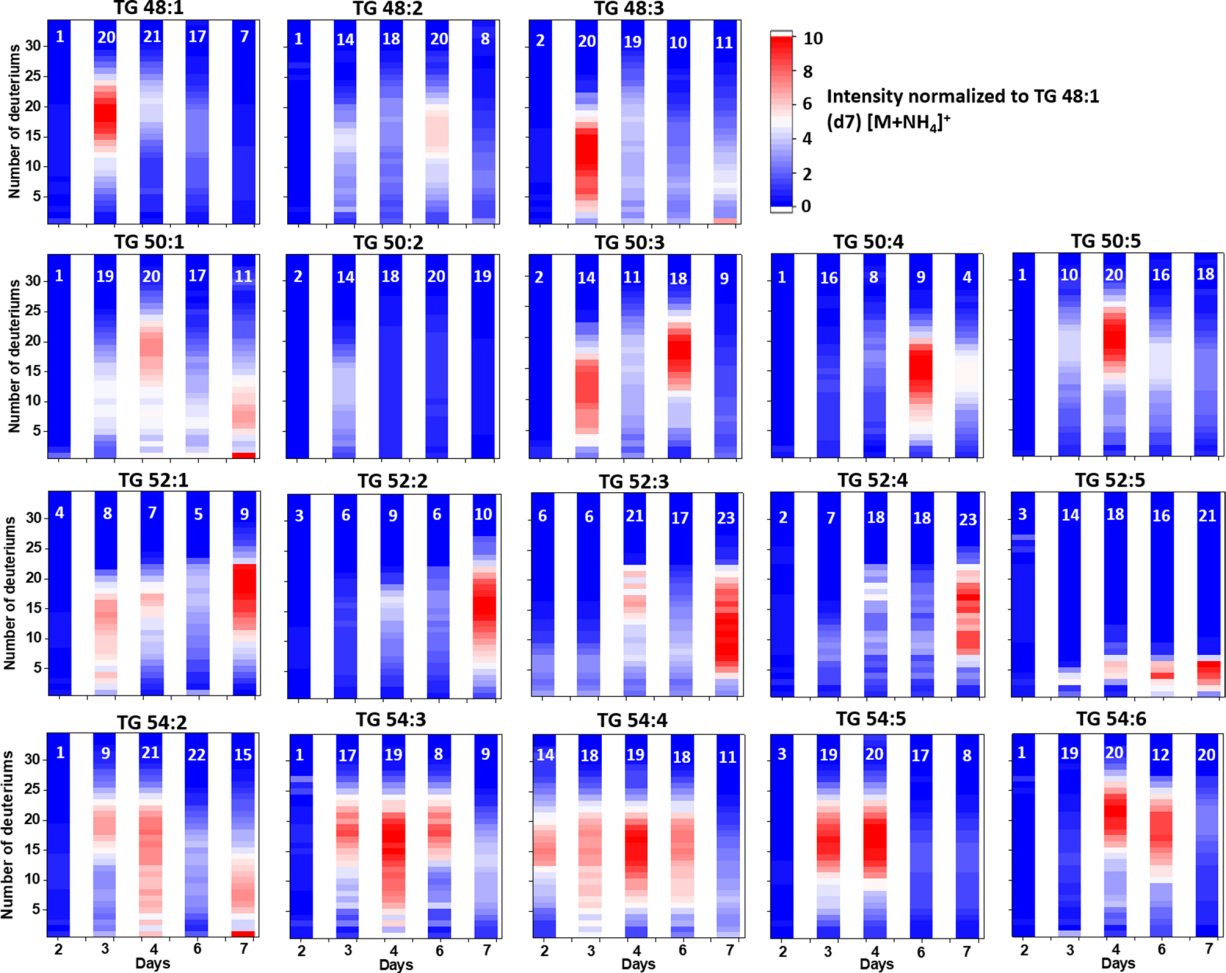
Distribution of the number of deuteriums per TG species as a function of days after eclosion (2–7 days). The median number of deuteriums is shown in the insets (white label). The color scale corresponds to the amount of deuterated species relative to the TG 48:1 (d7) internal standard.

The investigation of the dynamic changes of ovarian lipids during the PVG period was assisted by the incorporation of stable isotopes, providing an additional dimension of absolute quantitative values. The analysis of the number of deuterium atoms incorporated into eighteen different TG species at different days after adult eclosion, indicated a dynamic constant adjustment of ovarian TG stores during the experiment. In Fig. 5, we display the distribution of the number of deuterium incorporated into each TG as a function of time. Results are expressed as amount of ovarian TG deuterated species relative to the TG 48:1 (d7) internal standard using a color scale. The replacement of hydrogen by deuterium in TGs was already detected at day 2 after eclosion. At day 3 we observed a significant increase of ^2^H-TGs. The day when we recorded the highest number of incorporated ^2^H was different depending on the carbon number and unsaturation degree of the TG. At day 4, TGs 54:2, 54:5 and 54:6 have at least 20 × ^2^H incorporated into their lipid chains; while TG 50:4, TG 52:1 and TG 52:2 have less than 10 × ^2^H incorporated. We observed a positive correlation between the number of ^2^H incorporated into a TG and the carbon number, molecular size and number of unsaturated bonds. TG 54 and TG 52 were more abundant than TG 48, validating a relation between chain length and ability to incorporate deuterium. Interestingly, at day 7 the median number of ^2^H incorporations and total amount of TGs species in the ovary decreased; except for TGs 52:3, 52:4 and 52:5, suggesting that some of the follicles might have been resorbed. In terms of kinetics, the saturated species, such as TG 50:1; TG 52:1; TG 54:2, showed high deuterium incorporation at day 3, suggesting the deuteriums incorporated in single bonds are more stable than those incorporated in species rich in double bonds, such as TG 50:5; TG 52:5 and TG 54:6.

## Discussion

Oogenesis is energetically costly. *Ae. aegypti* females can lay over 120 eggs in a gonotrophic cycle; therefore, a tightly regulated control of nutrient allocations to the ovaries is critical^5,7,8,11,28^. The ovarian resting stage in *Ae. aegypti*, is a period marked by constant adjustment of the reproductive output based on nutritional and hormonal status; this adjustment typically occurs through follicular resorption by apoptosis^5,7,31^. The link between an increased oocyte lipid content and a successful reproductive output has been reported in invertebrate and vertebrate systems^12,14,24^. Teneral reserves are critical for the normal PVG development in *Aedes aegypti*. When teneral reserves are abundant, the CA is stimulated to synthesize JH and the maturation of primary follicles starts; otherwise, females need to increase their reserves by sugar-feeding, before maturation of the follicles is activated. Some insects totally depend on teneral reserves for reproduction and do not feed as adults. Some mosquito species are autogenic and are able to complete VG without a blood meal, and sometimes even without a sugar meal. By resorbing excess reproductive tissues, mosquitoes can alter previous reproductive decisions by redirecting resources away from reproduction in favor of competing physiological activities. Resorption during the ORS occurs in response to poor nutrition/starvation signals but can be reversed by JH administration. However, female mosquitos’ ability to mobilize/transfer nutrients out of the follicles, thus reversing previous reproductive allocations remains to be investigated. In this study, we started to address a fundamental question: “Can we use stable isotopes to establish time profiles of TGs incorporation into the developing ovarian follicles (composition, abundance and dynamic changes), which could lead us to a better understanding of the effects of nutrients on the dynamic importation of follicular lipids during oogenesis?”

In the current experiments we focused on addressing the issue of the distinct contributions of teneral reserves (those already carried at adult eclosion) and adult sugar feeding to the TGs imported into the ovarian follicles during the previtellogenic stage. To discriminate between these two nutritional sources, we labeled the lipids that were de novo synthesized using a ^2^H_2_O labeled sugar diet. Taking the advantage of stable isotope labelling; our studies confirmed that a significant portion of the ovary lipids were isotopically labeled with ^2^H, indicating an active de novo TG synthesis and transport into the ovaries. The dynamic changes on the ovarian TG lipid profiles suggested that ovarian TG lipids were utilized and replaced by de novo synthesized lipids. An increase of ^2^H-labelling was correlated with increases in carbon number, molecular size, and number of unsaturated bonds of TGs. Overall, the replacement of hydrogen atoms by deuterium is also influenced by enzymatic activities and kinetic parameters. Based on lipid dynamics, labeled ^2^H-TGs were incorporated into the ovaries during the entire experiment, but the total lipid amount was either reduced or stable by day 7, suggesting that some of the follicles might have been resorbed.

In summary, our studies showed de novo TG lipid mobilization and storage into the developing ovaries. The use of a stable isotope labeled-sugar diet enabled the dynamic study of lipid incorporation into ovarian follicles at the TG species level. The LC-UHRMS workflow provides the isotopic profile of characteristic TG species and the number of deuterium incorporated. Multiple TG species were detected in the 800 to 900 *m/z* range with a mass accuracy lower than 300 ppb. Non-labeled diets and labeled diets promoted similar quantities of TG lipids to be transferred to the ovaries. Despite the existence of a significant amount of lipid teneral reserves, TGs were synthetized de novo from the second day after adult emergence, and rapidly stored in the oocytes. An overall rise in de novo TG synthesis was observed for all TG species, with increases over time in the chain length sizes and number of unsaturated bonds of labelled TGs. The kinetics of label incorporation over time supports the idea that ovarian lipids are consumed or recycled during the PVG stage. Future studies using additional stable isotope (e.g., ^13^C) and complementary TG measurements from other mosquito tissue compartments (e.g., hemolymph, fat body, etc.) can provide further information about the TG allocation and mobilization in and out of the ovaries during the PVG stage.

## Methods

### Mosquito rearing conditions

*Aedes aegypti* of the Rockefeller strain were reared at 28 °C and 80% relative humidity. Male and female pupae were separated before adult eclosion. After eclosion, adult female mosquitoes were fed two different diet regimens: (a) 20% Sucrose/ultrapure water (HPLC); b) 20% Sucrose/heavy water (^2^H_2_O). During the experiments, insects were fed daily by wetting a 1 × 1 inch cotton pad with each individual diet. Containers were loosely covered with polyethylene wrap to prevent rapid evaporation of the feeding pad.

### Dissection and extraction of lipids

Female mosquitoes maintained on the two different diets were collected at different times after adult eclosion (2, 3, 4, 6 and 7 days) and immobilized by exposure to ice. Ovaries were dissected by performing an incision in the thorax, cutting the last abdominal segment and pulling out the ovaries^28^. Triplicates samples of 10 ovaries each were placed in 1.5 mL Eppendorf tubes, and 10 µL of a mix of labeled internal standard were added (EquiSplash Lipidomix, Avanti Polar Lipids, Alabaster, AL). After adding 100 µL of butanol/methanol and 3 µL of butylated hydroxytoluene, samples were homogenized for 10 s using polypropylene pestles (Fisher Scientific, Pittsburgh, PA), and a handheld cordless motor. The pestles were then rinsed with 200 µL of butanol/methanol. All tubes were sonicated for 30 min, and then centrifuged for 10 min. The supernatant was transferred into autosampler vials with 300 µL silanized glass inserts (Thermo Fisher Scientific, Waltham, MA).

### LC-FT-ICR MS analysis

Lipid identification and quantitation were performed at the National High Magnetic Field Laboratory (Florida State University, Tallahassee, FL), using a 14.5 T Fourier-transform ion cyclotron resonance Mass Spectrometer (FT-ICR-MS) equipped with a hexapolar detection (3Ω +) system. The instrument is a hybrid linear quadrupole ion trap/FT-ICR MS (LTQ-FT, Thermo Fisher Corp., Bremen, Germany), adapted to operate in an actively shielded 14.5 T superconducting magnet (Magnex, Oxford, U.K.). Analyses were performed in positive ion mode, using an electrospray ionization (ESI) source, operating at 3.9 kV capillary voltage and 325 °C capillary temperature. All raw data were processed using Thermo Xcalibur software (version 3.0.63) and a custom-built script using ZeroBrane Studio. The experimental MS resolving power was m/Δm_50%_ > 1,000,000 at *m/z* 800. The 14.5 T FT-ICR MS was coupled to a Waters e2695 Alliance HPLC system, equipped with an Accucore C_30_ column (Thermo Fisher Scientific, Sunnyvale, CA). Separations were done using acetonitrile (ACN), water (H_2_O) and isopropanol (IPA). The phase A was a gradient with 30:40:30 (ACN:H_2_O:IPA) and phase B was 10:5:85 (ACN:H2O:IPA). Both phases contained 10 mM ammonium acetate and 0.1% formic acid. HPLC conditions were an injection volume of 5 µL, solvent flow rate of 0.25 mL/min and 60 min of total run. The lipid intensity was normalized by the absolute intensity of the deuterated internal standard. The quantification was calculated based on the ratio between concentrations of the ovarian lipid species and internal standard. Standard error (± SE) was calculated between triplicate samples of 10 ovaries each.

## Supplementary Information

Supplementary Information 1.
