# Author Correction: Following de novo triglyceride dynamics in ovaries of *Aedes aegypti* during the previtellogenic stage

**DOI:** 10.1038/s41598-021-95696-y

**Published:** 2021-08-05

**Authors:** Lilian Valadares Tose, Chad R. Weisbrod, Veronika Michalkova, Marcela Nouzova, Fernando G. Noriega, Francisco Fernandez‑Lima

**Affiliations:** 1 Department of Chemistry and Biochemistry, Florida International University, 11200 SW 8th St AHC4‑233, Miami, FL 33199 USA; 2 National High Magnetic Field Laboratory, Florida State University, Tallahassee, FL USA; 3 Department of Biology, Florida International University, Miami, FL USA; 4 Biomolecular Science Institute, Florida International University, Miami, FL USA; 5 Institute of Parasitology, Biology Centre CAS, Ceske Budejovice, Czech Republic; 6 Institute of Zoology, Slovak Academy of Sciences, Dubravska cesta 9, 84506 Bratislava, Slovakia

Correction to: *Scientific Reports* 10.1038/s41598-021-89025-6, published online 05 May 2021

In the original version of this Article Veronika Michalkova was incorrectly affiliated with ‘Institute of Parasitology, Biology Centre CAS, Ceske Budejovice, Czech Republic’. The correct affiliations are listed below.

Department of Biology, Florida International University, Miami, FL, USA.

Institute of Zoology, Slovak Academy of Sciences, Dubravska cesta 9, 84506 Bratislava, Slovakia.

The original Article and accompanying Supplementary Information file have been corrected.

