# Supplementary material for: Following de novo triglyceride dynamics in ovaries of Aedes aegypti during the previtellogenic stage
Source: Sci Rep. 2021 May 5;11:9636. doi: 10.1038/s41598-021-89025-6 (PMC8099868; doi:10.1038/s41598-021-89025-6)
Supplement: Supplementary file 1 — Supplementary Information 1. [file 41598_2021_89025_MOESM1_ESM.pdf]

Following de novo triglyceride dynamics in ovaries of Aedes aegypti during the previtellogenic stage

Authors

Lilian Valadares Tose[1], Chad R. Weisbrod[2], Veronika Michalkova[3,6], Marcela Nouzova[3,4,5], Fernando G. Noriega[3,4], Francisco Fernandez-Lima[1,4*]

Affiliations

[1]Department of Chemistry and Biochemistry, Florida International University, Miami, FL, USA

[2]National High Magnetic Field Laboratory, Florida State University, Tallahassee, FL, USA

[3]Department of Biology, Florida International University, Miami, FL, USA

[4]Biomolecular Science Institute, Florida International University, Miami, FL, USA.

[5]Institute of Parasitology, Biology Centre CAS, Ceske Budejovice, Czech Republic.

[6]Institute of Zoology SAS, Bratislava, Slovakia.

* e-mail: fernandf@fiu.edu

Supplementary Information

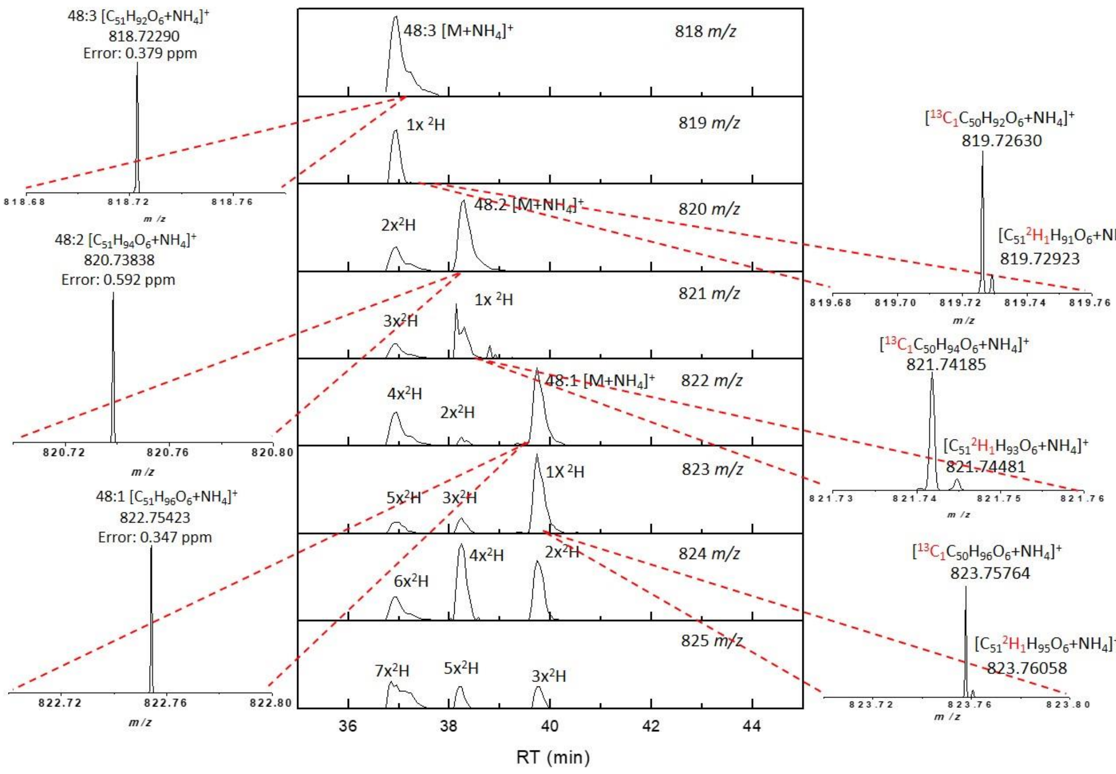

Supplementary Figure S1. Extracted ion chromatograms for TG 48:3, 48:2, and 48:1 from ovaries of females fed a deuterium labeled diet. Isotopic profiles for TGs are presented by highlighting $^2H/^{13}C$ in red.

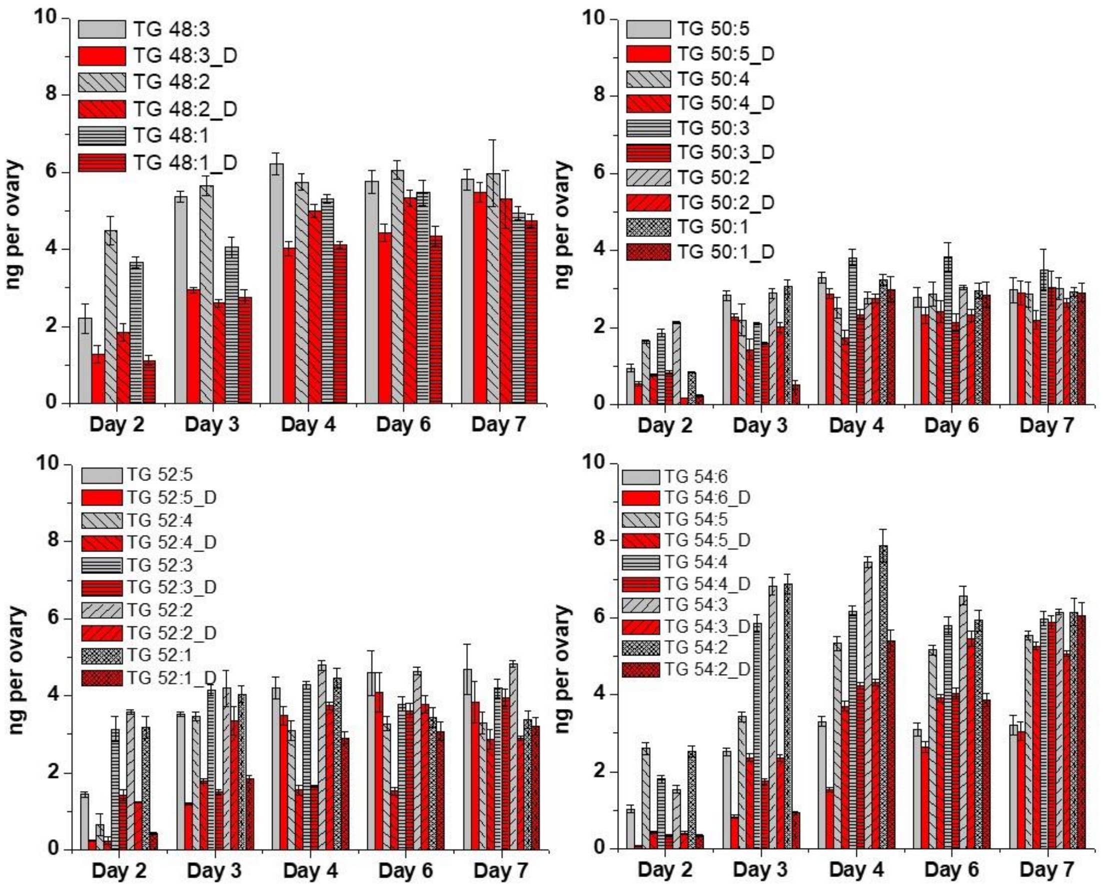

Supplementary Figure S2. Quantitation of total amount of the different TG species in ovaries as a function of diet (Normal or deuterated _D) and time in days.
